# Offensive Breath

**Published:** 1892-06

**Authors:** 


### OFFENSIVE BREATH.

Offensive breath, the bad odor exhaled by some persons when we approach them is common enough, and is often an unconscious nuisance, for, from a feeling of delicacy, one does not like to allude to it, and it may be imperceptible to the sufferer himself. Still, it is one of those minor miseries of life which we would be glad to be rid of.

And it exists in all degrees, from the slightest taint of a dyspeptic tongue to the horrible odor from carious bone and the stench of cancerous lung. In a large number of cases the teeth are in fault, and the dentist can best remove it ; but occasionally he is the unintentional cause from having neglected to warn a patient to always remove artificial teeth at bed-time and to very carefully cleanse them before replacement. The fætor due to the abuse of alcoholic drinks is very distinctive ; the excessive use of cider gives an odor like that of mustard, and the beer smell is but too well known to us all. If persistent, we can hardly be too careful or too unwearied in endeavoring to discover the cause ; the detection of the early stage of cancer of the gullet, of caries of the nasal bones, of diphtheria, of lung mischief of greater or less gravity, may reward the search. There is also the allied subject of the odor emitted by the healthy skin of the most scrupulously clean, and the singularity that it differs so widely not only in people of different nationalities, but in individuals. It is even more strange that, while we are painfully aware of it in others, we are quite unconscious of our own. When the mouth is in fault, rinsing it out with a warm but very weak solution of permanganate of potash—a single crystal in half a tumbler of water—frequently, is most effectual ; so is rinsing with a solution almost as dilute of tannic acid. And a thorough daily relief of the bowels is another precaution not to be overlooked.

